# A bovine lactoferricin-lactoferrampin-encoding *Lactobacillus reuteri* CO21 regulates the intestinal mucosal immunity and enhances the protection of piglets against enterotoxigenic *Escherichia coli* K88 challenge

**DOI:** 10.1080/19490976.2021.1956281

**Published:** 2021-08-07

**Authors:** Weichun Xie, Liying Song, Xueying Wang, Yigang Xu, Zengsu Liu, Dongfang Zhao, Shubo Wang, Xiaolong Fan, Zhaorui Wang, Chong Gao, Xiaona Wang, Li Wang, Xinyuan Qiao, Han Zhou, Wen Cui, Yanping Jiang, Yijing Li, Lijie Tang

**Affiliations:** a College of Veterinary Medicine, Northeast Agricultural University, Harbin, China; b Northeastern Science Inspection Station, China Ministry of Agriculture Key Laboratory of Animal Pathogen, Harbin, China

**Keywords:** newborn piglets, *Lactobacillus reuteri* CO21, lactoferricin-lactoferrampin (LFCA), Enterotoxigenic *Escherichia coli*

## Abstract

Enterotoxigenic *Escherichia coli* (ETEC) is an important cause of diarrhea in human and animal. To determine the mechanism of a bovine lactoferricin-lactoferrampin (LFCA)-encoding *Lactobacillus reuteri* CO21 (LR-LFCA) to enhance the intestinal mucosal immunity, we used a newborn piglet intestine model to study the intestinal response to ETEC. Pigs were chosen due to the anatomical similarity between the porcine and the human intestine.4-day-old piglets were orally administered with LR-LFCA, LR-con (*L. reuteri* CO21 transformed with pPG612 plasmid) or phosphate buffered saline (PBS) for three consecutive days, within 21 days after these treatments, we found that LR-LFCA can colonize the intestines of piglets, improve the growth performance, enhance immune response and is beneficial for intestinal health of piglets by improving intestinal barrier function and modulating the composition of gut microbiota. Twenty-one days after, piglets were infected with ETEC K88 for 5 days, we found that oral administration of LR-LFCA to neonatal piglets attenuated ETEC-induced the weight loss of piglets and diarrhea incidence. LR-LFCA decreased the production of inflammatory factors and oxidative stress in intestinal mucosa of ETEC-infected piglets. Additionally, LR-LFCA increased the expression of tight junction proteins in the ileum of ETEC-infected piglets. Using LPS-induced porcine intestinal epithelial cells (IPEC-J2) *in vitro*, we demonstrated that LR-LFCA-mediated increases in the tight junction proteins might depend on the MLCK pathway; LR-LFCA might increase the anti-inflammatory ability by inhibiting the NF-κB pathway. We also found that LR-LFCA may enhance the antioxidant capacity of piglets by activating the Nrf2/HO-1 pathway. This study demonstrates that LR-LFCA is effective at maintaining intestinal epithelial integrity and host homeostasis as well as at repairing intestinal damage after ETEC infection and is thus a promising alternative therapeutic method for intestinal inflammation.

## Introduction

Enterotoxigenic *Escherichia coli* (ETEC) is a major cause of diarrhea in man and animal. ETEC infections are the leading cause of travelers’ diarrhea and a major cause of diarrhea in developing nations, where it can be life-threatening among children.^1,2^ Gut microbes play important roles in host health and disease throughout life, particularly in infancy. The colonization of intestinal flora in infancy is a critical period for the formation of intestinal flora, which will affect the future growth and health of the body.^3^ The beneficial intestinal microflora not only helps in the digestion of food compounds but also reduces the potential of pathogen colonization in the guts.^4^ Many researchers have demonstrated that early intervention with desirable probiotics may help to establish a stable bacterial ecology and improve immunological development in the early life of human and animals.^5^ However, an important factor to consider is that probiotic properties are strain dependent, and it is not common to find microorganisms with multiple probiotic properties.^6^ Thus, using lactic acid bacteria (LAB) to produce the desired protein has become a new focus of research.^7,8^

Antimicrobial peptides (AMPs) are short cationic molecules (12–50 aa) with amphipathic structures, and these molecules play essential roles in host defense against microbial infection.^9^ Bovine lactoferricin (Lfcin B) and lactoferrampin (Lfampin) are two antimicrobial peptides released by gastric pepsin cleavage of bovine lactoferrin (LF).^10^ Lfcin B consists of a positively charged looped peptide containing residues 17–41, Lfampin comprises residues 268–284 in the N1 domain of LF.^11^ Recent reports have suggested that the fusion of Lfcin B with Lfampin (LFCA) broadens their antimicrobial spectra *in vitro*.^12,13^ In addition to its antimicrobial activity, the chimera has also been reported to be involved in improving performance, immune function and intestinal mucosal morphology.^14^ The synthesis and purification of AMPs are costly and time-consuming.^15^ *Lactobacillus* has been considered a good delivery vehicle to express AMPs for preserving the mucosal integrity, improving intestinal microbiota and ameliorating DSS-induced intestinal injury.^16,17^

In this sense, AMPs serves as a strategy to provide protection against ETEC colonization and infection.^18^ Despite an obvious clinical need, there are a number of barriers to the study of gut function and immune regulation. Studies on human infants with intestinal diseases continue to unavoidably suffer from the limitations of fecal analysis and remain confounded by variables with recognized impact on the intestinal bacterial community composition including differences in mode of delivery, gestational age, type of enteral nutrition, concurrent disease and treatment with antibiotics.^19^ Thus, many studies are based on animal models. Rodent models, although useful for molecular and cellular analysis, may not accurately reflect the physiology and pathophysiology of the human intestine. The pig is accepted as the best model for the study of human intestinal biology and diet.^20–22^ Its intestinal microbiota is similar to humans,^23^ and it is an ideal model for the study of complex gastrointestinal diseases.

In this study, we used the *Lactobacillus reuteri* (isolated from the intestinal mucous of a healthy piglet) as a mucosal delivery vehicle and developed a recombinant strain of *L. reuteri* secreting biologically active LFCA (LR-LFCA). 4-day-old piglets were selected as the model, to explore the effect of early intervention with LR-LFCA on animal health from the perspective of gut microbiota, intestinal physiological function and growth performance. We hypothesized that LR-LFCA would modulate the structure of the gut microbiota, improve the intestinal barrier, and promote healthy growth in infancy.

## Materials and methods

### Bacterial strains and growth conditions

*Lactobacillus reuteri* strains were isolated from the intestinal mucous of a healthy 35-day-old weaned piglet (Duroc × Landrace × Large White) and plated on *Lactobacillus* anaerobic MRS (de Mann Rogosa Sharpe; Oxoid, Hampshire, UK) contained with 1% CaCO_3_ according to the method described earlier.^24^ Isolates were characterized by Gram staining, catalase, and microscopic morphology observations. Molecular identification was utilized to identify the obtained strains. The total genomic DNA of isolates was extracted using the genomic DNA purification kit (TransGen Biotech Co., Ltd., Beijing, China), following the manufacturer’s instructions. The universal primers, 27 F and 1492 R, were used to amplify 16S rRNA genes: 27 F (5ʹ-AGAGTTTGATCMTGGCTCAG-3ʹ) and 1492 R (5ʹ -TACGGYTACCTTGTTAC GACTT-3ʹ),^25^ Enterobacterial repetitive intergenic consensus PCR(ERIC-PCR) was performed using the forward primer 5ʹ-ATGTAAGCTCCTGGGATTCAC-3ʹ and the reverse primer 5ʹ-AAGTAA GTGACTGGGGTGAGCG-3ʹ.^26^

The isolated *L. reuteri* were grown in MRS broth at 37°C. Genetically modified strains of *L. reuteri* (LR-LFCA) were grown in MRS supplemented with 10 μg/mL chloramphenicol. *Staphylococcus aureus* CVCC25923, enterotoxigenic *Escherichia coli* ETEC K88 (O8:H19: F4ac^+^, LT^+^, STa^−^, STb^+^), *Pasteurella multocida* ATCC43137 and *Salmonella enteritidis* ATCC50335 were cultured in LB (Oxoid, Hampshire, UK) broth for 12 hours to reach saturation (≥1.0 × 10^8^ colony-forming units (CFU) per mL).

### Construction of LR-LFCA

The two segments of Lfcin B and Lfampin were joined with a flexible linker (GGGS), called LFCA. The T7g10-sp-LFCA gene containing the sequence of T7g10 enhancer, signal peptide of peptidoglycan hydrolase and LFCA were synthesized and inserted into pUC57 vector by Genewiz Biological Technology Company, Ltd., Beijing, China (the sequences of gene encoding T7g10 enhancer, signal peptide of peptidoglycan hydrolase and LFCA were shown in Table S4). The plasmid, pMD-T7g10-sp-LFCA, was then digested by restriction enzymes *SnaB* I and *Apa* I (New England Biolabs) to generate compatible ends, T7g10-sp-LFCA transgene was ligated (T4 DNA ligase, New England Biolabs) into the pPG612 plasmid (which was kindly provided by Prof. Seegers, NIZO Institute, Netherlands) at the *SnaB* I and *Apa* I site. The map of recombinant plasmid pPG612-T7g10-sp-LFCA was shown in Figure S3. The pPG612-T7g10-sp-LFCA was then electrotransformed into competent *L. reuteri* CO21, which was further plated on MRS plates (with 10 μg/mL chloramphenicol) for positive selection as previously described,^27^ to generate pPG612-T7g10-sp-LFCA/LR CO21 (LR-LFCA).

Recombinant LFCA-expressing *L. reuteri* CO21 (LR-LFCA) and *L. reuteri* CO21 transformed with pPG612 plasmid (LR-con) were cultured in MRS broth supplemented with 10 μg/mL chloramphenicol for 24 hours. Cultures were centrifuged at 10,000 g for 5 minutes, and the cell supernatant and pellet were collected. The supernatant was treated with 10 mL of 100% trichloroacetic acid and incubated for 10 minutes on ice to precipitate proteins (a 20-fold concentration) as the method previously described.^28^ The supernatant protein precipitate and cell pellet were analyzed by 18% Tricine-SDS-PAGE and western blot assay were performed using an anti-bovine lactoferrin polyclonal antibody (prepared by our laboratory and diluted at 1:500) as the primary antibody and a horseradish peroxidase (HRP)-conjugated goat anti-mouse IgG (1:4000) as the secondary antibody. The concentration of secreted LFCA was quantitated using the principles previously described.^29^ Cell lysates and culture supernatant of *L. reuteri* CO21 transformed with pPG612 plasmid (referred as LR-con) were used as controls.

### Antibacterial activity assays of LFCA protein in vitro

The killing activity of the lysate of LR-LFCA was determined by a colony culture assay as described previously.^14^ Briefly, *S. aureus* CVCC25923, ETEC K88, *P. multocida* ATCC43137 and *S. enteritidis* ATCC50335 were grown in the presence or absence of bacterial lysate from LR-LFCA (5.0 μg of LFCA protein) in LB medium at 37°C for 6 h, 12 h, 18 h and 24 h; bacterial lysate from LR-con was used as a negative control. The incubated mixture was serially diluted in physiological saline and plated on LB agar. Colonies were counted after incubation at 37°C for 24 h. The percent killing or inhibition was calculated using the formula [1 − (CFU sample/CFU control)] × 100%. Each assay was performed on three separate occasions, each with triplicate determinations. After treatment with LFCA protein for 24 h, the *S. aureus* CVCC25923, ETEC K88, *P. multocida* ATCC43137 and *S. enteritidis* ATCC50335 cell pellets were treated as previously described,^12^ and observed using transmission electron microscopy with a Model JEM-1230 microscope (JEOL, Japan) operated at 100 kV.

### Animals and experimental design

Animal maintenance and experimental treatments were approved by the Animal Care and Use Committee of Northeast Agricultural University. The piglet study was conducted as previously described.^30^ 100 4-day-old piglets (Duroc × Large White × Landrace) were randomly allocated to 3 treatments within each litter were adjusted for an average body weight of 1.29 ± 0.15 kg and balanced gender. Each group was given different treatments as follows: (1) oral administration of 4 mL PBS to 4-day-old piglets from day 4 to day 6(CON). (2) oral administration of LR-con (5.0 × 10^9^ cfu mL^−1^ dissolved in 4 mL of PBS) to 4-day-old piglets from day 4 to day 6 (LR-con). (3) oral administration of LR-LFCA (5.0 × 10^9^ cfu mL^−1^ dissolved in 4 mL of PBS) to 4-day-old piglets from day 4 to day 6 (LR-LFCA). The piglets were individually housed in stainless steel cages (1.4 m × 0.45 m × 0.6 m). The environment was maintained at 30 ± 2°C and relative humidity (65–70%). Eight randomly selected piglets from each treatment were sacrificed after anesthesia by i.m. injection with sodium pentobarbital (40 mg kg-1 BW). Body weight was recorded on the morning of d 13, d 20 and d 27 to calculate the average daily gain (ADG).

Twenty-two days post the administration of LR-LFCA, the piglets weaned at 28 days of age. A corn–soybean meal basal diet was formulated to meet the nutrient requirements for the piglets according to the NRC,^31^ piglets were assigned to 4 groups (n = 6 per group) and challenged orally with a dose of 10^9^ CFU per piglet of the ETEC K88 for 5 days. The following four groups were as follows: (1) control (CON) group (six piglets from control treatment and oral administration of 10 mL sterile PBS only, from day 1 to 5); (2) ETEC K88 (ETEC) group (six piglets from control treatment and oral challenge with 1 × 10^8^ CFU ETEC K88 dissolved in 10 mL PBS on day 1 to 5); (3) ETEC + LR-con (ETEC+LR-con) group (six piglets from LR-con treatment and oral challenge with 1 × 10^8^ CFU ETEC K88 dissolved in 10 mL PBS on day 1 to 5); (4) ETEC + LR-LFCA (ETEC+LR-LFCA) group (six piglets from LR-LFCA treatment and oral challenge with 1 × 10^8^ CFU ETEC K88 dissolved in 10 mL PBS on day 1 to 5). Groups were kept physically separated by a solid partition; there was no direct contact between animals from different groups. Simultaneously, individual body weights were recorded daily. The attributed score for diarrhea was as follows: 0, normal; 1, loose stool; 2, loose/some diarrhea; 3, diarrhea; 4, severe watery diarrhea. Diarrhea score ≥2 indicated diarrheic feces whereas scores of diarrhea index <2 indicated absence of diarrhea. Diarrhea rate was calculated according to the following formula: diarrhea rate (%) = (number of piglets with diarrhea × diarrhea days)/ (number of piglets × total observational days) ×100 (Huang et al., 2004). On day 6 (5 days after ETEC K88 challenge), all piglets in each of the four groups were sacrificed.

### Samples collection

Samples of the middle jejunum and distal ileum were carefully collected, rinsed with PBS, and fixed in 4% paraformaldehyde. Other segments of the intestine tissue were opened and thoroughly rinsed with sterile normal saline, and then intestinal mucosa was collected. Some of the intestinal mucous was homogenized plated on MRS agar plates containing 10 μg/mL chloramphenicol. The plates were incubated overnight at 37°C for 48 h and colonies were counted to analyze the colonization ability of LR-LFCA in piglets. The remaining intestinal mucus was stored at −80°C. The blood samples and cecum contents of the piglets were collected into a vacuum tube, and the serum was collected after centrifugation at 2,000 × g for 15 min and stored at −80°C for further analysis.

### Intestinal morphology analysis and intestinal antioxidant parameters

Hematoxylin and Eosin (H&E) staining was performed to analyze intestinal morphology as described before.^32^ The villous height (VH) and crypt depth (CD) of each segment were measured with Image-Pro software (Media Cybernetics, Rockville, MD). A minimum of 8 villi from each sample were measured for each treatment as described previously.^33^

The mucosa samples of duodenum, jejunum and ileum were homogenized in ice-cold saline solution (1:4, wt/vol), and then the mucosal supernatant was prepared after centrifugation at 2500 × g for 10 min at 4 °C to determine the levels of intestinal antioxidant indexes in each section of small intestine. The intestinal mucosa antioxidant parameters including total antioxidant capacity (T-AOC), superoxide dismutase (SOD), malondialdehyde (MDA), catalase (CAT) and glutathione peroxidase (GSH-Px) were measured by the commercial kits (Nanjing Jiancheng Institute of Bioengineering, Jiangsu, China) with UV-VIS Spectrophotometer (UV1, 100, MAPADA, Shanghai, China) according to the manufacturer’s instructions.

### Microbiome analysis

Microbial DNA was extracted from piglets cecal samples using the E.Z.N.A. stool DNA Kit (Omega Biotek, USA) according to the manufacturer’s protocol. The V3-V4 region of the eukaryotic 16S rRNA gene was amplified using the primers 341 F (5ʹ-CCTACGGGNGGCWGCAG-3ʹ) and 806 R (5ʹ-GGACTACHVGGGTATCTAAT-3ʹ). Each amplified product was concentrated by solid-phase reversible immobilization and quantified by electrophoresis using a model 2100 Bioanalyzer (Agilent, USA). After quantifying the DNA concentration using a NanoDrop spectrophotometer, each sample was diluted to 1 × 10^9^ mol/µL in Tris-EDTA buffer and pooled. Then, 20 µL of the pooled mixture was sequenced with the MiSeq sequencing system (Illumina, USA) according to the manufacturer’s instructions. The resulting reads were analyzed as previously described.^34^

### Inhibitor treatments

The IPEC-J2 cells was kindly provided by Prof. Yanming Zhang, College of Veterinary Medicine, Northwest A&F University, Yangling, Shanxi, China. The cells were maintained in an incubator at 37°C in 5% CO_2_ in DMEM/F12 supplemented with 10% (v/v) fetal bovine serum (Gibco). Cells (1 ×10^5^ cells/well) were seeded in plastic six-well culture plates (Corning, Tewksbury, MA, USA) and maintained for 10 days (1 × 10^6^ cells/well). Confluent monolayers of IPEC-J2 cells were pretreated with 50 μM ML-7 (a selective inhibitor of MLCK) Sigma-Aldrich (St. Louis, MO) or vehicle (0.1% DMSO) for 6 h and then treated with bacterial lysate from LR-LFCA (10.0 μg of LFCA protein) or bacterial lysate from LR-con for 4 h before exposure to 1 μg/mL LPS for 12 h, then IPEC-J2 cells were collected for western blot analysis. Total cellular protein was extracted form tissue samples or cell lines using Cell lysis buffer for western and IP, and nuclear protein was extracted using the Nuclear Protein Extraction kit (Beyotime Biotechnology, Shanghai, China). The protein concentration was determined using a BCA Protein Assay Kit (Beyotime Biotechnology, Shanghai, China).

The porcine TLR-4 and MyD 88 gene siRNA interference vector was designed and synthesized by Shanghai Shenggong Biotechnology Co., Ltd. IPEC-J2 cells were plated on a 12-well plate, and after 12 hours of culture, 1 μg/mL siRNA was transfected with lipofectamine-3000 transfection reagent After transfection for 6 hours and washing with PBS for 3 times, add LPS 1 μg/mL to incubate the transfected cells for 24 hours. After washing 3 times with PBS, the cells were treated with lysis solution to extract total cell protein.

### ELISA, determination of mRNA expression

The concentrations of IL-1β, IL-12, IL-6, TNF-α, IL-10 and Secretory immunoglobulin A (SIgA) were measured using commercially available ELISA kits according to the manufacturer’s instructions (R&D Systems, Minneapolis, MN). The IgG level in serum samples were measured using commercially available swine ELISA kits (Shanghai Meilian Biological Technology Co., Ltd., Shanghai, China) according to the manufacturer’s instructions. The endotoxin and the D-lactic acid levels in the serum were measured using commercial ELISA kits (Nanjing Jian cheng Bioengineering Institute of China) and a commercial chromogenic end point Tachypleus kit (Xiamen Limulus Amebocyte Lysate Company, Xiamen, China), respectively, according to the manufacturer’s protocol.

The mRNA expression levels of ZO-1, Claudin-2, TLR4, Myd88 and MLCK were determined by real-time quantitative PCR. Total RNA was extracted from the jejunal and ileal tissue mucosa or IPEC-J2 cells using the TRIzol Plus RNA Purification Kit (Invitrogen, USA) following the manufacturer’s guidelines, and then reverse transcribed into cDNA using M-MLV reverse transcriptase (Promega). The cDNA was then analyzed by real-time quantitative PCR using iTaq SYBR Green Supermix (Bio-Rad, Hercules). The relative gene expression level was calculated using the comparative CT method (ΔΔCt method), and results were normalized to GAPDH housekeeping genes. The primers in the reaction were shown as supplement Table S5.

### Immunofluorescence staining

According to a previous method,^35^ IPEC-J2 cells were fixed in 4% paraformaldehyde, permeabilized with 0.1% Triton X-100, and blocked with 1% BSA. Then, the monolayers were incubated with primary antibodies against ZO-1 (1:50) or Claudin-2 (1:100) overnight at 4°C. The cells were subsequently incubated with the appropriate secondary fluorescent-conjugated antibody for 1 h. Cell nuclei were stained with DAPI. Fluorescence was examined by fluorescence microscopy using a ZOETM Fluorescent Cell Imager (Bio-Rad, Singapore).

### H_2_O_2_-IPEC-J2 cell oxidative damage experiment

LDH release rate detection: IPEC-J2 cells were seeded into 96-well culture plates at a density of 2 × 10^4^ cells/well, cultured for 12 h (cell density 80–90%), washed twice with PBS, and replaced with serum-free medium. Subsequently, LR-LFCA lysate (10 μg LFCA) or LR-con lysate was added for 4 h, and 0.5 mM H_2_O_2_ was added for 1 h. Centrifuge the cell culture plate at 400 g for 5 min in a multiwell plate centrifuge. Take 120 μL of the supernatant from each well and add them to the corresponding wells of a new 96-well plate, and then perform sample measurement. Add 60 μL of LDH detection working solution to each well, mix, and incubate at room temperature for 30 min in the dark. The absorbance was measured at 490 nm. After subtracting the absorbance of the background control from the absorbance of each well, calculate the LDH release rate of the cells according to the following formula.

LDH release rate (%) = (absorbance of processed sample-absorbance of sample control hole)/(absorbance of cell maximum enzyme activity-absorbance of sample control hole) × 100%

Reactive oxygen detection: IPEC-J2 cells were seeded into 6-well culture plates at a density of 5 × 10^5^ cells/well, cultured for 24 hours, and then pretreated with LR-LFCA lysate (10 μg LFCA) or LR-con lysate 4 h, add 0.5 mM H_2_O_2_ for 1 h. Dilute DCFH-DA with serum-free culture medium according to 1:1000 to make the final concentration 10 μmol/L. Remove the cell culture medium and add 1.5 mL of diluted DCFH-DA. Incubate for 30 minutes in a 37 °C cell incubator. Wash the cells three times with serum-free cell culture medium to fully remove the DCFH-DA that has not entered the cells. Process cell samples for flow cytometry.

### Isolation of porcine peripheral blood mononuclear-derived dendritic cells (MoDCs) and detection of cell surface molecules and phagocytic ability

The anterior vena cava blood of 4–7 weeks old piglets was collected with a vacuum blood collection tube. After the cells were incubated with anti-pig CD4-PE and CD172a-PE antibodies, immature dendritic cells and CD4^+^ T cells were screened out with anti-PE magnetic beads. Cultured immature dendritic cells for 6 days, stimulated with LPS (100 ng/mL), LPS and LR-LFCA lysate (10 μg LFCA protein) or LPS and LR-con lysate for 24 h, after the stimulated cells were collected into three parts. Part according to the ratio of MoDCs cell number: T cell number =1:4 was mixed into a 96-well plate, and the final volume of each well was 200 μL. Set T cell control wells, dendritic cell control wells, RPMI-1640 culture medium control wells, three parallel samples for each group, culture in a 37 °C, 5% CO_2_ incubator for 3 d, add CCK-8 dye 10 μL/ Measure the OD_450_ value with a microplate reader and calculate the stimulation index (SI) of T cells according to the following formula: SI = (DC+T)OD_450_﹣(DC) OD_450_/(T)OD_450_﹣ (medium)OD_450_.

Part of MoDCs cells were seeded in a 96-well plate at a cell density of 1 × 10^5^, 100 μL of 0.1% neutral red was added to each well, and the cells were collected and cultured in a 37 °C, 5% CO_2_ incubator. After 2 hours, they were discarded and not phagocytosed. Wash the neutral red with preheated PBS twice, then add 100 μL 1% SDS lysate to each well, let the cells be lysed at room temperature for 2 hours, set 3 replicate wells in each group, and finally read on the microplate reader Take the OD_540_ value, the OD value is proportional to the phagocytic function of the cell. The remaining MoDCs were incubated with anti-MHC-II-FITC and anti-CD40-FITC at 4°C for 30 minutes. Detect the expression level of cell surface molecules by flow cytometry or real-time PCR.

### Statistical analysis

Mean ± SD values were calculated, and the statistical significance of differences was analyzed using one-way analysis of variance (ANOVA) followed by multiple comparisons between groups using Tukey’s post-hoc test. Differences with *p* values less than 0.05 were considered significant, and the significance is reported as *p ≤ 0.05 and **p ≤ 0.01. All calculations were performed using SPSS 19.0 software.

## Results

### Generation of a L. reuteri CO21 strain producing LFCA, and bioactivity of LFCA

As observed microscopically, the *L. reuteri* strains isolated from the intestinal mucous were gram-positive, catalase-negative, rod-shaped bacteria (Figure S1A). CE1, CO21, CE12 and J31 were all identified as *L. reuteri* by ERIC-PCR and 16 s rRNA gene sequencing with 99% similarity (Figure S1B, C, Table S1). We gained 4 *L. reuteri* isolates from the piglet intestinal mucous. Further analysis of isolates showed *L. reuteri* CO21 had a highest capability to resistance to lysozyme, bile, low pH (pH 2.0 and 3.0) as shown in Table S2. The isolate *L. reuteri* CO21 also showed the highest percentage of hydrophobicity and auto-aggregation rate (Table S3). So we used *L. reuteri* CO21 as a mucosal delivery vehicle. The *L. reuteri* CO21 had been kept in the China Center for Type CultureCollection (Preservation number: CCTCC NO: M2019601).

**Figure 1.**
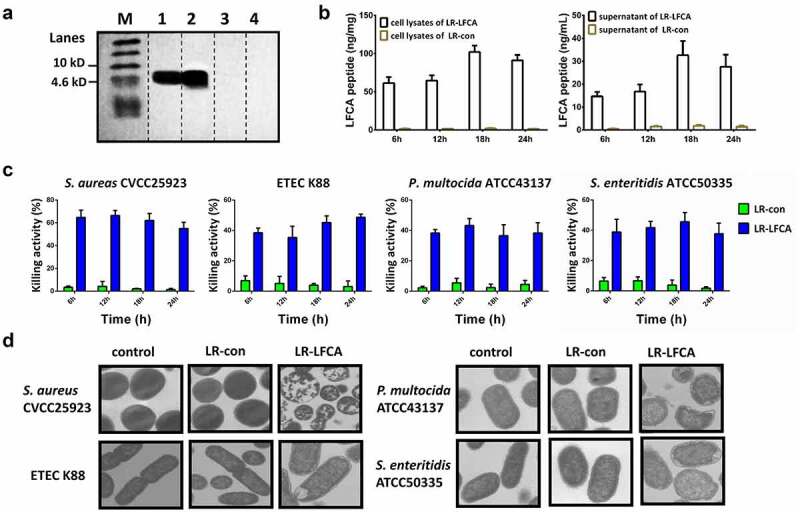
Characterization of the recombinant *L. reuteri* CO21 strain producing LFCA (LR-LFCA) and LFCA produced by LR-LFCA is bioactive. Protein production and secretion were analyzed by western blotting, which resolved LFCA as an immunoreactive 4.6 kDa band (panel A), and ELISA (panels B). LR-LFCA and LR-con were cultured for 24 h, and LFCA levels were assayed in the cell lysates and 20-fold TCA-concentrated culture supernatant. 1: The culture supernatant of LR-LFCA, 2: the cell lysates of LR-LFCA, 3: the culture supernatant of LR-CON, 4: the cell lysates of LR-CON. Dotted lines separated the lanes, and irrelevant lanes were omitted. (c) Bar graph showing the effects of lysates (5 µg protein) from LR-LFCA cells on the growth of *S. aureus* CVCC25923, ETEC K88, *P. multocida* ATCC43137 and *S. enteritidis* ATCC50335. (d) Ultrastructural damage in bacteria treated with cell lysates (5 µg protein) from LR-LFCA. Control, bacteria treated with PBS; LR-con, bacteria treated with cell lysates from LR-con; LR-LFCA, bacteria treated with cell lysates from LR-LFCA. Cells were analyzed by electron microscopy. Data are presented as the means ± SD of three independent replicates

In this study, we engineered recombinant *L. reuteri* CO21 strains with a plasmid expressing LFCA (LR-LFCA). Immunoblotting of LR-LFCA cell lysates and supernatant confirmed the expression and secretion of LFCA by the detection of a 4.6 kDa band (Figure 1a). The expression level of LR-LFCA was highest in culture medium at 18 h (Figure 1b). The capacity of LR-LFCA cell lysates to inhibit the growth of *S. aureus*, ETEC K88, *P. multocida* and *S. enteritidis* strains was determined. LR-LFCA lysates, but not the LR-con lysates, significantly inhibited the growth of *S. aureus, E. coli* K88, *P. multocida* and *S. enteritidis* (55.07%, 48.65%, 38.31% and 37.79%, respectively,) when cultured with the lysate for 24 h (Figure 1c). Negative staining and electron microscopy revealed that LR-LFCA cell lysates damaged the morphology of *S. aureus, E. coli* K88, *P. multocida* and *S. enteritidis*, and bacteria treated with LR-LFCA lysate contained more atypical vesicles, protrusions, and filamentations than untreated cells (control) and cells treated with LR-con cell lysate (LR-con; Figure 1d).

### Oral administration of LR-LFCA to neonatal piglets improved the intestinal morphology and intestinal epithelial barrier

**Figure 2.**
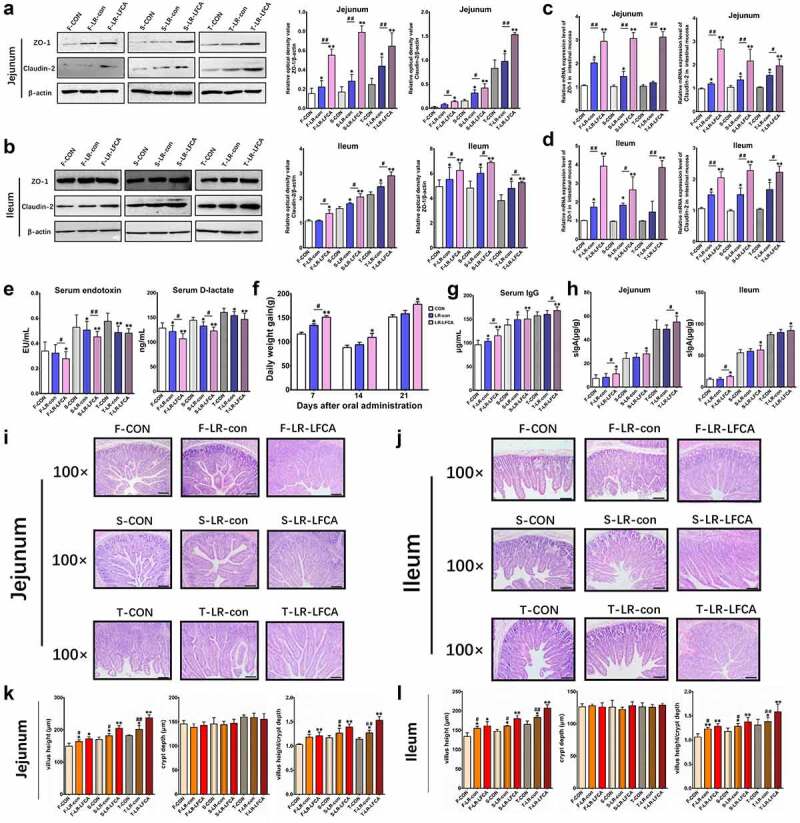
The effect of oral administration LR-LFCA on the intestinal morphology and the intestinal epithelial mucosa. (a, b) Western blot measurements of the expression levels of tight junction proteins (including ZO-1 and Claudin-2) in the jejunum and ileum of piglets. (c, d) Relative mRNA expression levels of ZO-1 and Claudin-2 in the jejunum and ileum mucosa determined by Realtime-PCR. (e) The serum D-lactic acid and endotoxin levels of piglets. (f) Growth performance of the piglets. (g) The levels of serum IgG. (h) The level of secretory IgA in jejunum and ileum. (i, j) Representative images of the jejunum and ileum stained with H&E (scale bar, 100 µm). (k, l) The crypt depth, villous height and villus height/crypt depth of the jejunum and ileum were measured. F-CON, the first week after oral administration of PBS; F-LR-con, the first week after oral administration of LR-con; F-LR-LFCA, the first week after oral administration of LR-LFCA; S-CON, the second week after oral administration of PBS; S-LR-con, the second week after oral administration of LR-con; S-LR-LFCA, the second week after oral administration of LR-LFCA; T-CON, the third week after oral administration of PBS; T-LR-con, the third week after oral administration of LR-con; T-LR-LFCA, the third week after oral administration of LR-LFCA. Data are represented as mean ± SD, *p < .05, **p < .01 vs. the CON group; #p < .05 and ##p < .01 vs. the LR-LFCA group

Data concerning the localization of the LR-LFCA in sections of gastrointestinal tract tissue is presented in Figure S2. Compared with CON group, colonization of the LR-LFCA in the duodenum, jejunum, ileum, cecum and colon was significantly increased at 7 days after administration. At 21 days after administration, the abundance of LR-LFCA in the duodenum, cecum and colon was not significantly different compared to that of CON group. However, at 21 days after administration, LR-LFCA still colonized the jejunum and ileum, and the abundance was significantly more than that in the CON group. The expression of TJ-associated proteins ZO-1 and Claudin-2 in jejunum and ileum were monitored and compared between groups using real-time PCR. Compared with CON group, the mRNA expression levels of ZO-1 and Claudin-2 were significantly increased (*p* < .01 or *p* < .05) relative to those in piglets treated with LR-LFCA or LR-con, LR-LFCA group was better than LR-con group (Figure 2 C, D). To further evaluate the effect of LR-LFCA on tight junction structure, we assessed the protein levels of ZO-1 and Claudin-2 by western blotting. As shown in Figure 2a, B, almost the same trends were observed. The levels of serum D-lactic acid and endotoxin, which are key indexes for intestinal epithelial barrier function evaluation were compared.^32^ D-lactic acid and endotoxin concentrations in LR-LFCA and LR-CON groups were significantly decreased compared with the CON group at days 7, 14 and 21 post-LR-LFCA administration (Figure 2e).

The effects of oral administration of LR-LFCA to neonatal piglets on the growth performance of piglets is presented in figure 2f. After oral administration of LR-LFCA to neonatal piglets, the average daily gain (ADG) was significantly increased, throughout the experiment. The ADG of LR-con treated piglets also showed significantly increased than CON piglets during the first week of the experiment, but not during the second and third week. As shown in Figure 2g, at days 7, 14 and 21 post-LR-LFCA administration, the levels of serum IgG were effectively increased compared with the CON group, throughout the experiment. The levels of serum IgG in LR-con group was significant increased compared with the CON group after 7 days, 14 days, but not after 21 days. Compared with the CON group, the levels of secretion of IgA in jejunum and ileum were significantly increased in the LR-LFCA group, at days 7, 14 and 21 post-LR-LFCA administration (Figure 2 H). Compared with the CON group, the levels of intestinal secretory IgA in the LR-con group showed no significant statistical difference. As shown in Figure 2 I-L, at days 7, 14 and 21 post-LR-LFCA administration, the piglets fed with LR-LFCA and LR-con significantly increased villi height (VH) and the ratio of villus to crypt (VH:CD) in the middle jejunum and distal ileum parts, compared to those of CON group, but the LR-LFCA group had better effect on VH and VH:CD than LR-con group. The crypt depth did not differ among groups.

### Oral administration of LR-LFCA to neonatal piglets improved the gut microbiota of piglets

**Figure 3.**
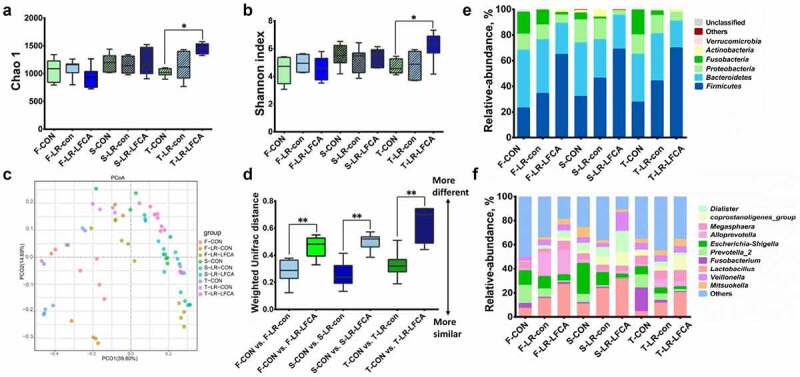
The changes of intestinal microbial diversities in piglets with oral administration of LR-LFCA. (a) Chao 1 index. (b) Shannon index. (c) Principal coordinates analysis (PCoA) of the operational taxonomic units in the piglet cecum contents. (d) β-Diversity between groups was analyzed by weighted UniFrac distance. Microbial community bar plot at the (e) phylum level and (f) genus level. F-CON, the first week after oral administration of PBS; F-LR-con, the first week after oral administration of LR-con; F-LR-LFCA, the first week after oral administration of LR-LFCA; S-CON, the second week after oral administration of PBS; S-LR-con, the second week after oral administration of LR-con; S-LR-LFCA, the second week after oral administration of LR-LFCA; T-CON, the third week after oral administration of PBS; T-LR-con, the third week after oral administration of LR-con; T-LR-LFCA, the third week after oral administration of LR-LFCA. Data are presented as mean ± SD. *p < .05, **p < .01 vs. CON; ^#^*p* < .05 and ^##^*p* < .01 vs. the LR-LFCA group

The microbiota composition and diversity of the cecal contents at days 7, 14 and 21 post-LR-LFCA administration was assessed by deep sequencing of the V3-V4 region of the 16S rRNA genes. The relative abundance of cecal microbiota in piglets at the phylum and genus levels were displayed in Figure 3e and F. *Bacteroidetes* and *Firmicutes* were the most dominated phyla in piglets, followed by *Proteobacteria*, and *Fusobacteria* (Figure 3e). LR-LFCA treatment increased the proportions of *Firmicutes*, and decreased the proportion of *Bacteroidetes, Proteobacteria* and *Fusobacteria* compared with those in the CON group at days 7, 14 and 21 post-LR-LFCA administration. LR-con treatment also increased the proportions of *Firmicutes* but did not decrease the proportion of *Bacteroidetes* and *Proteobacteria* when compared with the CON group during the three period. At the genus level, during the three period, administration of LR-LFCA decreased the relative abundances of the *Escherichia–Shigella, Fusobacterium* and *Prevotella_2* population and increased the relative proportion of *Lactobacillus*, whereas the other major families were only marginally affected when compared with CON group (figure 3f). Administration of LR-con also enhanced the relative proportion of *Lactobacillus* and decreased the relative abundances of the *Fusobacterium* and *Prevotella_2* population but did not decrease the relative proportion of the *Escherichia–Shigella* population when compared with the CON group. Collectively, the above results indicate that administration of LR-LFCA to newborn piglets significantly changes the intestinal microbiota composition of piglets. Alpha diversity was analyzed by calculating the Chao1 and Shannon indices (Figure 3a, b). The Simpson and Chao1 index showed negligible differences between the three groups after 7 days or 14 days administration, while the Simpson and Chao1 index were higher for the LR-LFCA group than for the CON and LR-con group, after 21 days administration. Beta diversity, the principal coordinate analysis (PCoA) based on weighted-unifrac distance revealed that the gut microbiota showed obvious segregation among treatments at days 7, 14 and 21 post-LR-LFCA administration (Figure 3c). The box-and-whisker plot of microbial communities in the CON, LR-con and LR-LFCA groups during three period was determined based on the weighted 16S UniFrac distances, and the result clearly showed that the microbial beta-diversity in the LR-LFCA group was greater than that in the other groups, and the difference was statistically significant (Figure 3d).

### Oral administration of LR-LFCA to neonatal piglets improved intestinal morphology and increased the expression of intestinal tight junction proteins of piglets after ETEC K88 challenge

**Figure 4.**
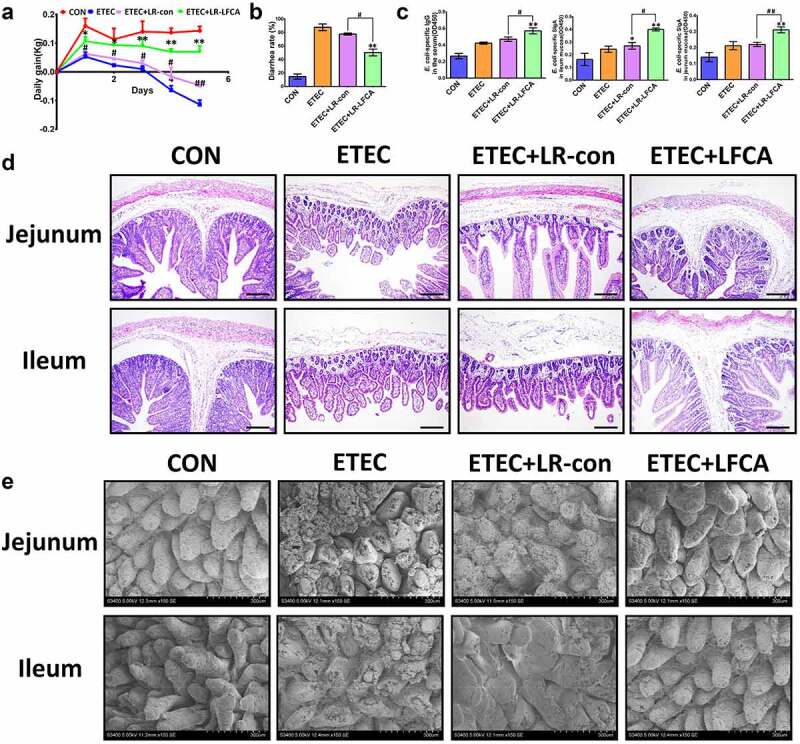
Effect of oral administration LR-LFCA to newborn piglets on intestinal morphology after ETEC K88 challenge. (a) Bodyweight changes of piglets. (b) Diarrhea incidence of piglets. (c) The concentration of ETEC-specific IgG in serum and ETEC-specific SIgA in jejunum and ileum mucosa. (d, e) Intestinal morphology of jejunum and ileum shown by H&E staining and scanning electronic microscope. Data are presented as mean ± SD. *p < .05, **p < .01 vs. CON; ^#^*p* < .05 and ^##^*p* < .01 vs. the ETEC＋LR-LFCA group

The body weight effects of oral administration of LR-LFCA on neonatal piglets after ETEC K88 challenge are presented in Figure 4a. A lower weight loss was observed in LR-LFCA piglets significantly after ETEC K88 challenge. Compared to the ETEC and ETEC＋LR-con groups, the ETEC＋LR-LFCA piglets exhibited lower rates of diarrhea (Figure 4b). To assess the immune responses to the oral administration of LR-LFCA in newborn piglets, we quantified the concentrations of IgG and SIgA in serum and intestine mucosa, respectively (Figure 4c). Compared to the ETEC group, LR-LFCA marked higher *E. coli*-specific IgG in serum and *E. coli*-SIgA levels in jejunum and ileum mucosa. Morphological analyses revealed that ETEC infection led to inflammatory infiltration and intestinal injury in the jejunum and ileum tissues of piglets, compared with the ETEC group. Oral administration of LR-LFCA to neonatal piglets attenuated ETEC-induced inflammatory infiltration and improved the structure of the intestinal mucosa (Figure 4d, e). However, there was no obvious difference between ETEC and ETEC＋LR-con group.

**Figure 5.**
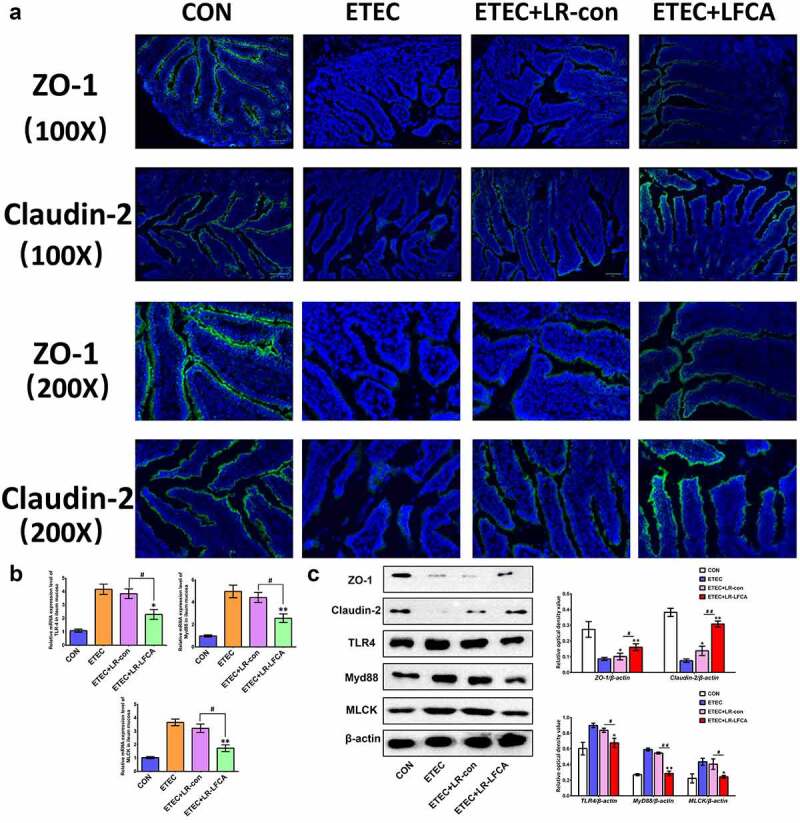
The expression of intestinal tight junction proteins after ETEC K88 challenge. (a) Immunofluorescence staining for ZO-1 and Claudin-2 in ileum of piglets. (b) The relative mRNA expression of TLR-4, Myd88, and MLCK in ileum of piglets, detected using real-time PCR. (c) The contents of ZO-1, Claudin-2, TLR-4, Myd88, and MLCK in ileum of piglets were determined by Western blot analysis. Data are presented as mean ± SD. *p < .05, **p < .01 vs. CON; ^#^*p* < .05 and ^##^*p* < .01 vs. the ETEC＋LR-LFCA group

We evaluated the effects of oral administration of LR-LFCA to neonatal piglets on epithelial physical intestinal barrier in the ileum by detecting TJ protein expression. The immunofluorescence results showed that ETEC infection decreased the protein expression of ZO-1 and Claudin-2 in the ileum (figure 4f). Compared with the ETEC group, the ETEC＋LR-LFCA group had increased protein expression of ZO-1 and Claudin-2 in the ileum, whereas the ETEC＋LR-con group had increased protein expression of only Claudin-2. The Western blot results confirmed that LR-LFCA attenuated the ETEC-induced decreases in ZO-1 and Claudin-2 protein expression in the ileum (Figure 5a). The level of TLR4, Myd88 and MLCK in ETEC-infected ileum of piglets was examined. We found the LR-LFCA could down-regulate the mRNA expression of TLR4, Myd88, and MLCK significantly in the ileum of ETEC-infected piglets (Figure 5b). However, ETEC K88 infection followed by LR-con treatment did not affect the TLR4, Myd88 and MLCK expression in the ileum of piglets. The Western blot results also showed that LR-LFCA decreased the protein expression of TLR4, Myd88 and MLCK in the ileum (Figure 5c).

### Oral administration of LR-LFCA to neonatal piglets protected against ETEC K88-induced oxidative stress and inflammatory factors changes in intestinal mucosa

**Figure 6.**
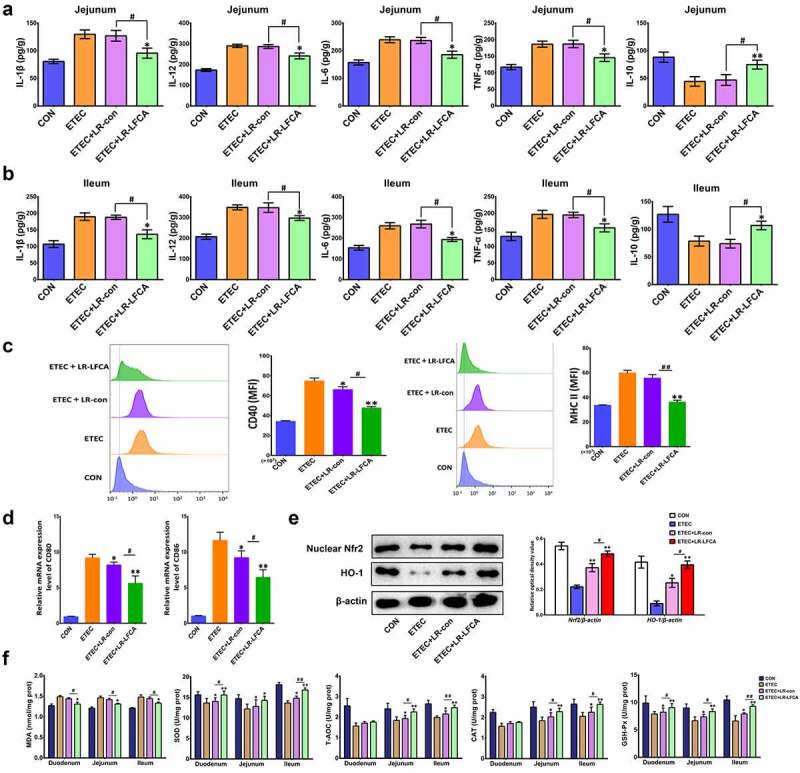
Effects of oral administration LR-LFCA to newborn piglets on oxidative stress and inflammatory factors changes in intestine mucosa of piglets after ETEC K88 challenge. (a) IL-1β, IL-12, IL-6, TNF-α and IL-10 levels in jejunum; (b) IL-1β, IL-12, IL-6, TNF-α and IL-10 levels in ileum, all of them were determined by ELISA. (c) The expression of CD40 and MHC II in dendritic cells in Peyer’s patch of piglets (d) The transcription levels of dendritic cells CD80 and CD86 in Peyer’s patch of piglets (e) Nuclear Nrf2 and HO-1 levels were assessed by western blotting. (f) MDA production; SOD activity changes; T-AOC activity changes; CAT activity changes; GSH-Px activity changes. Data are presented as mean ± SD. *p < .05, **p < .01 vs. ETEC; ^#^*p* < .05 and ^##^*p* < .01 vs. the ETEC＋LR-LFCA group

Pro-inflammatory cytokines IL-1β, IL-12, IL-6 and TNF-α levels in jejunum and ileum increased markedly and anti-inflammatory cytokine IL-10 levels decreased significantly in ETEC-treated piglets compared with the CON group. LR-LFCA significantly suppressed IL-1β, IL-12, IL-6 and TNF-α levels in jejunum and ileum. As expected, the administration of LR-LFCA also marked upregulation of IL-10 levels (Figure 6a-b). No differences in the levels of inflammatory cytokines were detected between the ETEC＋LR-con group and the ETEC group.

After ETEC K88 infection, the expression of CD40 and MHC-II on the surface of DC cells in the PP nodes of piglets increased significantly. Compared with the ETEC group, after oral administration of LR-LFCA or LR-con in lactating piglets, the expression of CD40 and MHC-II on DC cells in the PP nodes of piglets was significantly reduced, and the effect of the ETEC+LR-LFCA group was better than that of the ETEC+LR-con group (Figure 6c). Real-time PCR detection revealed that after ETEC K88 infection, the CD80 and CD86 gene transcription levels on the surface of DC cells in the PP nodes of piglets increased significantly. Compared with the ETEC group, the CD80 and CD86 gene transcription levels on the DC cell surface of the PP nodes of the piglets in the ETEC+LR-con group and ETEC+LR-LFCA group were significantly reduced, and the effect of the ETEC+LR-LFCA group was better than that of the ETEC+LR-con group (Figure 6d).

figure 6f presents the enzyme activities in the small intestine of weaned pigs. ETEC challenge increased the abundance of MDA in the small intestinal mucosa compared with CON group. Relative to ETEC group, administration of LR-LFCA decreased MDA level in the intestinal mucosa. The enzymatic activities of SOD, T-AOC, CAT and GSH-Px were also detected, and the levels of these antioxidase activities were also inhibited with ETEC K88 challenge. Oral administration of LR-LFCA to neonatal piglets significantly improved the levels of these antioxidase activities, but LR-con group had no significant effect on these indicators compared to the ETEC group. The changes in the expression of Nrf2 and HO-1 were examined by western blotting (Figure 6e). ETEC K88 challenge obviously decreased accumulation of Nrf2 and HO-1. However, the negative effect by ETEC K88 was attenuated in the ETEC＋LR-LFCA group. LR-con also increased the expression of Nrf2 and HO-1, but not as well as LR-LFCA.

### The mechanism of LR-LFCA regulating intestinal mucosal immunity

**Figure 7.**
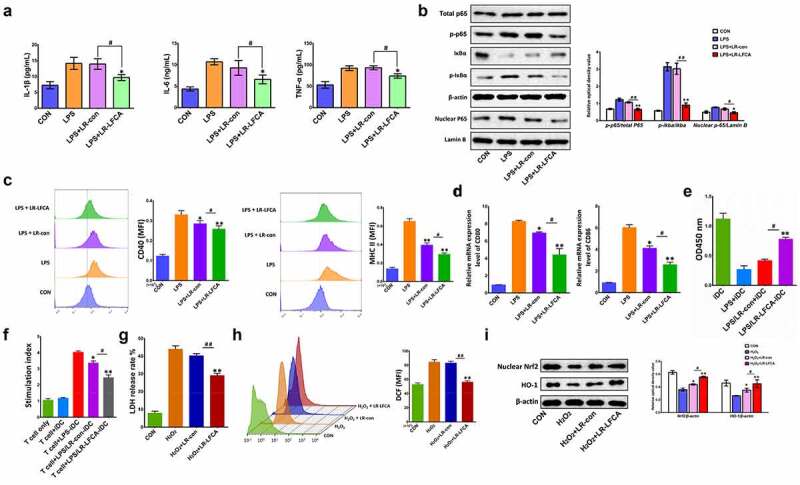
The anti-inflammatory and antioxidant mechanism of LR-LFCA. (a) IL-1β, IL-6 and TNF-α levels in LPS-induced IPEC-J2 cells were determined by ELISA. (b) Western blot analysis of p65, *p*-p65, IκB-α, *p*-IκB-α, lamin B, and β-actin. (c) CD80 and CD86 levels on the surface of LPS-induced MoDCs cells were determined by flow cytometry. (d) The mRNA expression of CD80 and CD86 on the surface of LPS-induced MoDCs cells were determined by Real-time PCR Data are means ± SD. (e) The MoDCs phagocytic ability was determined by neutral red. (f) The ability of LPS-stimulated MoDCs to mediate CD4 + T cell proliferation was determined by CCK-8. (g) The release rate of LDH in IPEC-J2 cells stimulated by H_2_O_2_. (h) The contents of reactive oxygen species in H_2_O_2_-stimulated IPEC-J2 cells were determined by flow cytometry, and the average fluorescence intensity of DCF were analyzed. (i) Western blot analysis of the protein expression levels of Nrf2 and HO-1 in IPEC-J2 cells. *p < .05, **p < .01 vs. the CON group; ^#^*p* < .05 and ^##^*p* < .01 vs. the LR-LFCA group

To determine the anti-inflammatory effects of LR-LFCA on LPS-induced IPEC-J2 cells, the expression of the inflammatory cytokines IL-1β, IL-12, IL-6 and TNF-α was evaluated via ELISA. Compared with the CON group, higher protein expression levels of IL-1β, IL-6, and TNF-α were detected in the LPS group. As expected, the administration of LR-LFCA could ameliorate that effect (Figure 7a). NF-κB plays a pivotal role in regulating cytokines. To explore whether the inhibition of inflammation by LR-LFCA is mediated by NF-κB pathway, NF-κB p65 and IκB phosphorylation levels were determined. The results showed that the phosphorylation of p65 and IκBα, and the nuclear translocation of NF-κB, were significantly enhanced in LPS group, but reversed in LR-LFCA treatment groups (Figure 7b).

Compared with the LPS group, LFCA treatment significantly reduced the overexpression of the surface molecules CD40 and MHC-II on MoDCs stimulated by LPS, and the phagocytic ability of MoDCs was significantly increased (Figure 7c-e). After LPS-stimulated MoDCs and CD4 + T cells were co-cultured, the proliferation ability of CD4 + T cells was significantly increased, while LR-LFCA and LR-con significantly reduced the proliferation of CD4 + T cells induced by LPS-stimulated MoDCs (figure 7f).The LFCA expressed by LR-LFCA alleviated the LDH release of IPEC-J2 cells and the increase in the fluorescence intensity of DCF caused by H_2_O_2_ stimulation, indicating that LFCA expressed by LR-LFCA can alleviate the cell oxidative damage caused by H_2_O_2_ stimulation to a certain extent (Figure 7g-h). Compared with the control group, the expression levels of nuclear transcription factors Nrf2 and HO-1 decreased significantly after H_2_O_2_ stimulation. Compared with the H_2_O_2_ group, the expression levels of the transcription factors Nrf2 and HO-1 in the H_2_O_2_+ LR-LFCA group and the H_2_O_2_+ LR-con group were significantly increased, and the effect of the H_2_O_2_+ LR-LFCA group was better than that of the H_2_O_2_+ LR-con group (Figure 7i).

**Figure 8.**
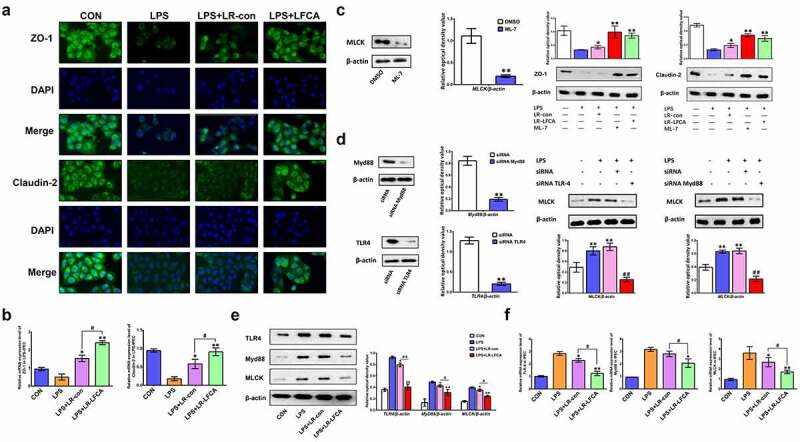
The effects of LR-LFCA on LPS-induced barrier dysfunction in vitro. (a) Immunofluorescence localization of ZO-1 and Claudin-2 in IPEC-J2 cells. (b) The mRNA expression of ZO-1 and Claudin-2 assessed by real-time PCR. (c) Content of ZO-1 and Claudin-2 in IPEC-1 cells with ML-7 treatment. (d) Content of MLCK in IPEC-J2 cells with siRNA-induced knock-down of TLR4 and MyD88. (e) The contents of TLR-4, Myd88, and MLCK in IPEC-J2 cells were determined by Western blot analysis. (f) The relative mRNA expression of TLR-4, Myd88, and MLCK in IPEC-J2 cells, detected using real-time PCR. Data are presented as mean ± SD. *p < .05, **p < .01 vs. CON; ^#^*p* < .05 and ^##^*p* < .01 vs. the LPS＋LR-LFCA group

Furthermore, we investigated the effects of LR-LFCA on the TJ expression in intestinal epithelial cells in vitro and the underlying mechanism. We found that LR-LFCA increased the protein expression of ZO-1 and Claudin-2 (Figure 8a-b). MLCK is a key protein that regulates tight junction protein expression. The content of MLCK protein in IPEC-J2 cells was suppressed by the selective inhibitor ML-7. It was then demonstrated that the increases in the content of ZO-1 and Claudin-2 caused by ML-7 or LR-LFCA in LPS-IPEC-J2 cells (Figure 8c). In order to explore the mechanism by which LPS regulates MLCK protein expression, this study used siRNA interference technology to silence the TLR4 and MyD88 genes in IPEC-J2 cells. The results indicate that LPS may increase the expression of TLR4 and MyD88 proteins, thereby causing the increase of MLCK protein expression, and then reducing the expression of tight junction proteins (Figure 8d). Based on the above findings, we further explored the mechanism by which LR-LFCA alleviates the LPS-induced reduction of cellular tight junction protein. We found that LR-LFCA decreased the protein expression of TLR4, Myd88 and MLCK in LPS-IPEC-J2 cells (Figure 8e-f).

Collectively, the above results indicated that oral administration recombinant LR-LFCA expressing LFCA significantly increased the expression of tight junction proteins ZO-1 and Claudin-2, correlating with the barrier function of intestinal epithelium, which may be mediated by TLR-4/MyD88 signal-transduction pathway up-regulation of the MLCK transcription and translation process. Alternatively, orally immunized neonatal piglets with LR-LFCA expressing LFCA can protect against ETEC K88-induced oxidative stress by activating the Nrf2/HO-1 pathway, and substantially improve the anti-inflammatory ability by inhibiting the NF-κB pathway, DCs maturation and T cell activation and proliferation.

## Discussion

Due to the physiological similarity between the human and porcine GI tract, pigs have not only been shown to be susceptible to many enteric human pathogens,^36–41^ but present symptoms characteristic of human infections.^42^ For instance, the newborn piglet model would enable studies on how the exposure to various components of the infant’s microbiome affects development of the innate immune system. Recent studies have shown a role of intestinal commensals in the development of mucosal immunity in neonates. The beneficial effects of LFCA expressed by recombinant *Lactococcus lactis* have been documented in our previous studies, including promoting the growth of piglets, improving the immunity of piglets, and alleviating the colonic tissue damage induced by DSS in mice.^31,43^ *L. reuteri* is one of the most well documented probiotic species and has been widely utilized as a probiotic in human for many years.^44^ It is widely distributed in the animal intestines and colonizes in both small and large intestines.^45,46^ This study provides the first comprehensive description of the intestinal mucosal immunity regulated by LFCA expressed by *Lactobacillus reuteri*. In this study, we isolated 4 *L. reuteri* from the piglet intestinal mucosa. *L. reuteri* is a probiotic bacterium resistant to acid, lysozyme, and bile and has strong hydrophobicity and auto-aggregation activity. These characteristics enable them to adapt to the harsh conditions of the gastrointestinal tract (GIT) and colonize the intestine.^47^ By testing these probiotic characteristics, we obtained a *L. reuteri* strain (*L. reuteri* CO21) with the best probiotic effect. Then we used *L. reuteri* CO21 as a delivery vehicle and successfully constructed the LFCA expressed recombinant *L. reuteri –* pPG612-T7g10-sp-LFCA/LR CO21 (LR-LFCA). The bioactivity of the recombinant *L. reuteri* was evaluated by testing its antimicrobial intensity.

**Figure 9.**
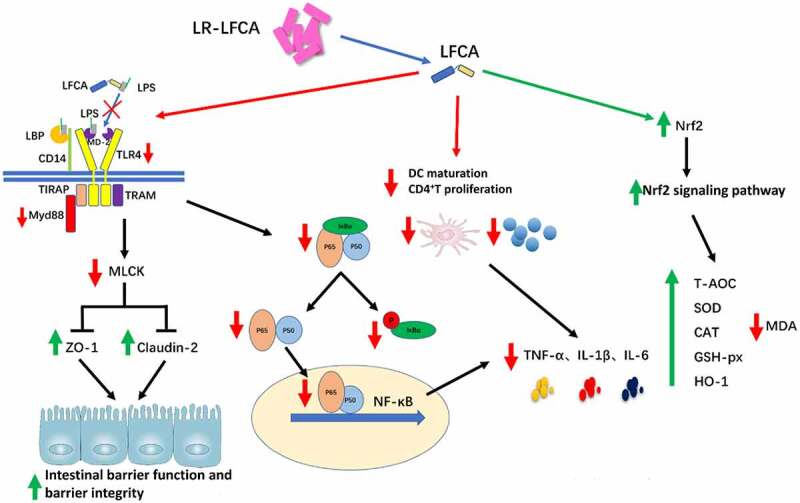
LR-LFCA regulates intestinal mucosal immune mechanism

IgG and IgA constitute the major antibody component in blood and extracellular fluid, respectively. They are the main immunoglobulins involved in humoral immunity.^48^ Our data demonstrated that LR-LFCA significantly increased serum IgG levels and intestinal sIgA levels. These data revealed that the humoral immunity level of piglets was improved by LR-LFCA in Figure 9. Intestinal morphology reflected in villus height (VH) and the ratio of villus height to crypt depth (VH:CD) plays an essential role in nutrient absorption as well as providing a protective barrier.^49,50^ Our study demonstrated that oral administration of LR-LFCA to neonatal piglets had better effect on VH and VH:CD in the jejunum and ileum compared to CON and LR-con group, which were beneficial to intestinal function.

The degradation of the intestinal barrier can provide a beneficial environment for the proliferation of the pathogen and potentiate more severe disease symptoms.^51^ The function of the intestinal barrier is related to many factors, including D-lactate, endotoxins in serum, and intestinal epithelial tight junction (TJ) proteins.^52,53^ Elevated levels of D-lactate in serum are usually accompanied by impaired function of the intestinal barrier.^54^ As one of the *E. coli* secretions, the escalated abundance of endotoxins could lead to enhanced intestinal permeability and poor intestinal barrier function.^55^ In the present study, lower serum concentrations of D-lactate and endotoxins were observed in LR-LFCA group compared with CON and LR-con group. ZO-1, Occludin and Claudins are major intestinal barrier proteins.^53^ Our data also demonstrated that oral administration of LR-LFCA to neonatal piglets significantly increased intestinal epithelial tight junction proteins (ZO-1 and Claudin-2) expression.

Previous studies have demonstrated that infancy is a key period of colonization of gut microbial flora.^56^ Moreover, the intervention with sodium butyrate in the first week can change the microbiota composition in piglets.^48^ Therefore, we selected the 4 d after birth as the intervention stage in our present study. The results obtained in this study clearly showed that 4 day after birth, piglets were given oral administration of LR-LFCA for three consecutive days significantly alters intestinal microbiota composition of piglets. In the first and second week after oral administration of LR-LFCA had no difference in richness of species among groups, in the third week after oral administration of LR-LFCA markedly enhanced α-diversity of gut microbiota evidenced by the increased Chao1 and Shannon indices. Meanwhile, the results of PCoA analysis and the intercommunity β-diversity determined by weighted 16S UniFrac distances indicated a significant difference among the gut microbial composition of the three groups dietary during each experimental period. Our analyses also showed that after oral administration of LR-LFCA to 4-day-old piglets decreased the percentage of *Bacteroidetes, Proteobacteria* and *Fusobacteria* and increased the percentage of *Firmicutes*. After oral administration of LR-LFCA, the abundance of *Escherichia–Shigella, Fusobacterium* and *Prevotella_2* was significantly reduced, and the relative proportion of *Lactobacillus* was enhanced. After oral administration of LR-con also enhanced the relative proportion of *Lactobacillus* and decreased the relative abundances of the *Fusobacterium* and *Prevotella_2* population but did not decrease the relative proportion of the *Escherichia–Shigella* population when compared with the CON group. These data indicate that oral administration of LR-LFCA to 4-day-old piglets promotes the proliferation of beneficial bacteria and reduces the proliferation of harmful bacteria, thus regulating the microbial barrier function.

Previous studies have shown that ETEC infection altered TJ protein expression and increased intestinal permeability in the intestine of piglets,^57^ consistent with this finding, we found that ETEC K88 challenge decreased the protein expression of ileum ZO-1 and Claudin-2. However, oral administration of LR-LFCA to neonatal piglets alleviated the damage caused by subsequent infection with ETEC K88. Similarly, LR-LFCA increased ZO-1 and Claudin-2 expression in LPS-induced IPEC-J2 cells. The up-regulate of claudin-2 expression is an important host defense response that limits intestinal pathology. In the absence of pore pathway water efflux, increased unrestricted pathway permeability allows diarrhea to develop.^58^ These data suggest that LR-LFCA increases TJ protein expression and regulates the physical intestinal barrier. To elucidate the mechanism of LR-LFCA regulating the TJ protein expression, the TLR-4, Myd88 and MLCK transcript and protein expression were detected. Our results suggested that the ETEC K88-induced reduction in TJ protein was because of an increase in expression of MLCK protein. Our data also showed that the ETEC K88-induced increase in MLCK expression and intestinal TJ protein was mediated by TLR-4/MyD88 signal-transduction pathway up-regulation of the MLCK transcription and translation process. The protective effect of LR-LFCA on LPS-induced intestinal barrier disruption was further confirmed in an in vitro model using the IPEC-J2 cell line. These results were consistent with a previous study by Nighot M et al.^59^

Oxidative stress is a pivotal factor that leads to intestinal damage, resulting from the overproduction of MDA and the decrease in antioxidant defense.^60^ In the present study, we observed that ETEC K88 challenge caused intestinal oxidative injury, indicated by an increase in the levels of MDA and a decrease in the activities of SOD, GSH-Px, CAT and T-AOC. However, oral administration of LR-LFCA to neonatal piglets alleviated these indicators, indicating LR-LFCA could improve the antioxidant capacity of weaned piglets. Nuclear factor (erythroid-derived 2)-like 2 (Nrf2) is a key regulator of cellular resistance to oxidative stress.^61^ Additionally, the up-regulation of HO-1 is mediated by Nrf2 and the activation of Nrf2/HO-1 pathway can protect cells from oxidative stress-induced damage.^62^ This study showed that oral administration of LR-LFCA to neonatal piglets increased the expression levels of Nrf2 and HO-1 in the ileum of weaned pigs. These combined findings collectively demonstrated that the enhanced activities of antioxidant enzymes mediated by Nrf2/HO-1 signaling pathway, might be the partial reasons why LR-LFCA could improve the antioxidative capacity of weaned pigs.

In a normal state, DCs have a weak response to inflammation and also produce a small amount of anti-inflammatory factors, and maintain intestinal immune homeostasis by promoting and maintaining the number of Treg cells.^63^ However, in an abnormal inflammatory state, the function of DCs will change, mature DCs are migrated to lymph nodes, and the expression of inflammatory factors increases to activate the initial T cells and cause a cascade of inflammation.^64^ Studies have found that biologically active peptides derived from milk can effectively inhibit bone marrow-derived dendritic cells (BMDCs) caused by LPS stimulation by reducing the expression of pro-inflammatory factors, the expression of surface molecules, and the ability of antigen presentation. Maturation and T cell proliferation/activation level, thereby alleviating intestinal inflammation.^65,66^ The study also found that ETEC K88 infection, piglets ileal Peyer’s the maturity of DCs significantly increased. The oral administration of LR-LFCA in lactating piglets significantly reduced the expression of CD40 and MHC II on the surface of DCs and the transcription level of CD80 and CD86 in the ileal Peel’s knot after weaned piglets were infected with ETEC K88. In addition, we also showed for the first time that LFCA inhibits the activation and proliferation of CD4 + T cells mediated by LPS stimulated by MoDCs, which may be one of the reasons for its inhibition of inflammation.

Cytokines play important roles in the inflammatory response and participate in the regulation of integrity of the intestinal barrier.^67,68^ IL-1β, IL-12, IL-6, TNF-α are markers of inflammatory response in intestine, whereas IL-10 is an anti-inflammatory master regulator in the intestine.^57^ Accumulating evidence has confirmed that ETEC K88 can induce intestinal barrier disruption of piglets by up-regulating the expression levels of pro-inflammatory cytokines.^69^ Consistent with these findings, results obtained here showed that ETEC K88 induced a profound increase IL-1β, IL-12, IL-6, TNF-α expression and decrease IL-10 expression that may cause disruption of the intestinal barrier, and oral administration of LR-LFCA to neonatal piglets may alleviate the intestinal damage by suppressing the over release of intestinal pro-inflammatory cytokines in weaned piglets. These results were in agreement with that LR-LFCA inhibited the increased expression of IL-1β, IL-6 and TNF-α induced by LPS in IPEC-J2 cells. To further explore the molecular mechanism of LR-LFCA in attenuating intestinal inflammatory response, the NF-κB signaling pathway was examined. When IPEC-J2 cells are stimulated by external stimuli, NF‐κB kinases can phosphorylate the IκB family of proteins, followed by ubiquitination and degradation, further phosphorylating p65 and releasing it into the nucleus, where it promotes inflammation.^70^ Western blot analysis showed that the phosphorylation of p65 and IκBα, which are initiated the activation of NF-κB in IPEC-J2 cells, was significantly increased by LPS and decreased by treatment with lysate from LR-LFCA cells, but not from LR-control cells. The findings also revealed that the anti-inflammatory effects of LR-LFCA may be related to the inhibition of the nuclear transfer of p65 NF-κB. These data suggesting LFCA expressed by LR-LFCA may reduce the production of pro-inflammatory factors by suppressing NF-κB-mediated transcriptional activation of inflammatory gene expression. This research focuses on the mechanism of LR-LFCA enhancing intestinal mucosal immunity. In future studies, we will explore whether the effect of ETEC depends on any toxin and is reversed by LR-LFCA.

In summary, this study constructed a recombinant *Lactobacillus reuteri* named LR-LFCA expressing bovine lactoferrin peptide, which could enhance the integrity of the piglet’s intestinal mucosal barrier function, increase the immunoglobulin content and improve the piglet’s intestinal tract Micro-ecological environment. After piglets were infected by ETEC K88, LR-LFCA could increase the expression of tight junction proteins ZO-1 and Claudin-2 by inhibiting the expression of TLR4, Myd88 and MLCK protein, and regulated the physical intestinal barrier. In addition, LR-LFCA could not only inhibit the NF-κB pathway, DCs maturation, T cell activation and proliferation to improve the anti-inflammatory ability of piglets, but also increased antioxidative capacity by activating Nrf2/HO-1 pathway. This may be the reason why oral administration of LR-LFCA to newborn piglets can increase the ability of piglets to resist ETEC K88 infection. This study provides a theoretical basis for the application of a bovine lactoferricin-lactoferrampin-encoding *Lactobacillus reuteri* CO21 as a microecological agent associated with strong infant immunity and optimal development.

## Supplementary Material

Supplemental Material

## Data Availability

All 16S rRNA sequencing data were submitted under accession No. SRP260545.
