# Supplementary material for: A bovine lactoferricin-lactoferrampin-encoding Lactobacillus reuteri CO21 regulates the intestinal mucosal immunity and enhances the protection of piglets against enterotoxigenic Escherichia coli K88 challenge
Source: Gut Microbes. 2021 Aug 7;13(1):1956281. doi: 10.1080/19490976.2021.1956281 (PMC8354667; doi:10.1080/19490976.2021.1956281)

A bovine lactoferricin-lactoferrampin-encoding *Lactobacillus reuteri* CO21 regulates the intestinal mucosal immunity and enhances the protection of piglets against enterotoxigenic *Escherichia coli* K88 challenge

Weichun Xie^a^, Liying Song^a^, Xueying Wang^a^, Yigang Xu^a,b^, Zengsu Liu^a^, Dongfang Zhao^a^, Shubo Wang^a^, Xiaolong Fan^a^, Zhaorui Wang^a^, Chong Gao^a^, Xiaona Wang^a,b^, Li Wang^a,b^, Xinyuan Qiao^a,b^, Han Zhou^a,b^, Wen Cui^a,b^, Yanping Jiang^a,b^, Yijing Li^a,b^, Lijie Tang^a,b^*

^a College of Veterinary Medicine, Northeast Agricultural University, Harbin, China^

^b Northeastern Science Inspection Station, China Ministry of Agriculture Key Laboratory of Animal Pathogen^

^*^ Corresponding author

Lijie Tang. Chang Jiang Road No. 600, Xiang Fang District, Harbin, China; Tel.: 86-451-55190824; Fax: 86-451-55190363; e-mail: tanglijie@163.com or tanglijie@neau.edu.cn

Table S1. The molecular identification results of *Lactobacillus reuteri* based on 16 S rRNA gene sequencing

| Lab isolates | Genera/species | Isolate accession  numbers |
| --- | --- | --- |
| *L. reuteri* CE1 | *Lactobacillus reuteri* | MK920163 |
| *L. reuteri* CO21 | *Lactobacillus reuteri* | MK920155 |
| *L. reuteri* CE12 | *Lactobacillus reuteri* | MK920992 |
| *L. reuteri* J31 | *Lactobacillus reuteri* | MK921700 |

Table S2. In-vitro screening of selected microbiota as candidate for probiotic

| Lab isolates | Lysozyme  Resistance (%) | pH tolerance (%) | | Bile salt tolerance (%) | |
| --- | --- | --- | --- | --- | --- |
|  |  | 2.0 | 3.0 | 0.1 | 0.3 |
| *L. reuteri* CE1 | 88.30 ±8.12 ^a^ | 68.22±10.57^a^ | 80.42 ±11.45^a^ | 67.34±7.57^a^ | 44.54±11.36^a^ |
| *L. reuteri* CO21 | 96.89 ±6.36^b^ | 77.19 ±8.63^b^ | 87.36 ±8.67^b^ | 71.42±8.36^a^ | 55.73±13.64^b^ |
| *L. reuteri* CE12 | 85.82 ±6.75^a^ | 49.57 ±9.47^c^ | 64.16 ±8.35^c^ | 32.65±6.92^b^ | 12.63±4.32^c^ |
| *L. reuteri* J31 | 71.32 ±5.71^c^ | 52.46 ±7.21^c^ | 66.78 ±5.60^c^ | 56.45±7.73^c^ | 29.73±9.51^d^ |

For each determination, different superscript letters in the same column indicate statistical differences in each strain at the level of *P* < 0.05 as measured by Tukey’s test, the values are represented as mean SD of three independent replicates.

Table S3. Hydrophobicity and auto-aggregation activity of isolates

| Lab isolates | Hydrophobicity (%) | Auto-aggregation (%) | |
| --- | --- | --- | --- |
|  |  | 3 h | 24 h |
| *L. reuteri* CE1 | 40.31±6.24^a^ | 23.43±5.69^a^ | 36.91±5.47^a^ |
| *L. reuteri* CO21 | 43.85±3.41^b^ | 28.38±3.22^b^ | 46.97±4.94^b^ |
| *L. reuteri* CE12 | 29.81±6.55^c^ | 10.74±2.57^b^ | 14.06±2.48^c^ |
| *L. reuteri* J31 | 58.43±2.85^d^ | 12.59±2.75^b^ | 16.23±2.72^c^ |

For each determination, different superscript letters in the same column indicate statistical differences in each strain at the level of *P* < 0.05 as measured by Tukey’s test, the values are represented as mean SD of three independent replicates.

Table S4. The sequences of gene encoding T7g10 enhancer, signal peptide of peptidoglycan hydrolase and LFCA

| Name of the sequence | the sequences of gene (5’-3’) |
| --- | --- |
| T7g10 | GGGAGACCACAACGGTTTCCCACTAGAAATAATTTTGTTTAA  CTTTAAGGAAAAAAGGAGCTGGAACCGATG |
| signal peptide of peptidoglycan hydrolase | ATGAAATTAAAGCAATTAGTTACAGGCTTTATCACAGTTGCAACATTGGCTGGGGTTGGGGTTTCAGGGGTGGCTGCCACAACAGTTAAAGCT |
| LFCA | TTTAAATGCCGTCGTTGGCAATGGCGCATGAAGAAGTTGGGCGCTC  CGAGTATTACCTGCGTTCGCCGCGCTTTTGGCGGTGGCTCCAGTGTTGATGGCAAAGAAGATTTGATTTGGAAATTGTTGAGTAAAGCTCAAGAAAAATTTGGCAAAAATAAAAGTCGT |

Table S5. Primers for Real-time quantitative PCR

| Gene | Primer Sequences | Accession number |
| --- | --- | --- |
| ZO-1 | F: 5’-AGCCCGAGGCGTGTTT-3’  R: 5’-GGTGGGAGGATGCTGTTG-3’ | XM_013993251 |
| Claudin-2 | F: 5’-GCTGGCGAACGAGTTCTTAC-3’  R: 5’-AGATGGCGCTAGATGTCACC-3’ | NM_001161638.1 |
| TLR-4 | F: 5’-AGGACGAAGACTGGGTG-3'  R: 5’-CTGGGCAATCTCATACTC-3’ | NM_001113039.2 |
| Myd88 | F: 5’-CCGTCGGATGGTAGTGG-3'  R: 5’-CAGTGATGAACCGCAGGAT-3’ | NM_001099923.1 |
| MLCK | F: 5’-CGGTTATCATCCGAAGC-3'  R: 5’-GTCCTGGAAGTCTGCCTC-3’ | XM_021070234.1 |
| GAPDH | F: 5’-ACTCACTCTTCCACTTTTGATGCT-3’  R: 5’-TGTTGCTGTAGCCAAATTCA-3’ | NM_001206359 |

Figure S1. The morphological characteristics of *lactobacillus reuteri* after gram staining were observed by oil lens. (A) 1: *L. reuteri* CE1; 2: *L. reuteri* CO21; 3: *L. reuteri* CE12; 4: *L. reuteri* J31. ERIC-PCR fingerprints of *L. reuteri* isolates (B). Lane M1, M2 contain the molecular size marker. lane 1: *L. reuteri* CE1; lane 2: *L. reuteri* CO21; lane 3: *L. reuteri* CE12; lane 4: *L. reuteri* J31. PCR amplification of 16S rRNA (C). Lane M contains the molecular size marker. lane 1: negative control; lane 2: *L. reuteri* CE1; lane 3: *L. reuteri* CO21; lane 4: *L. reuteri* CE12; lane 5: *L. reuteri* J31.


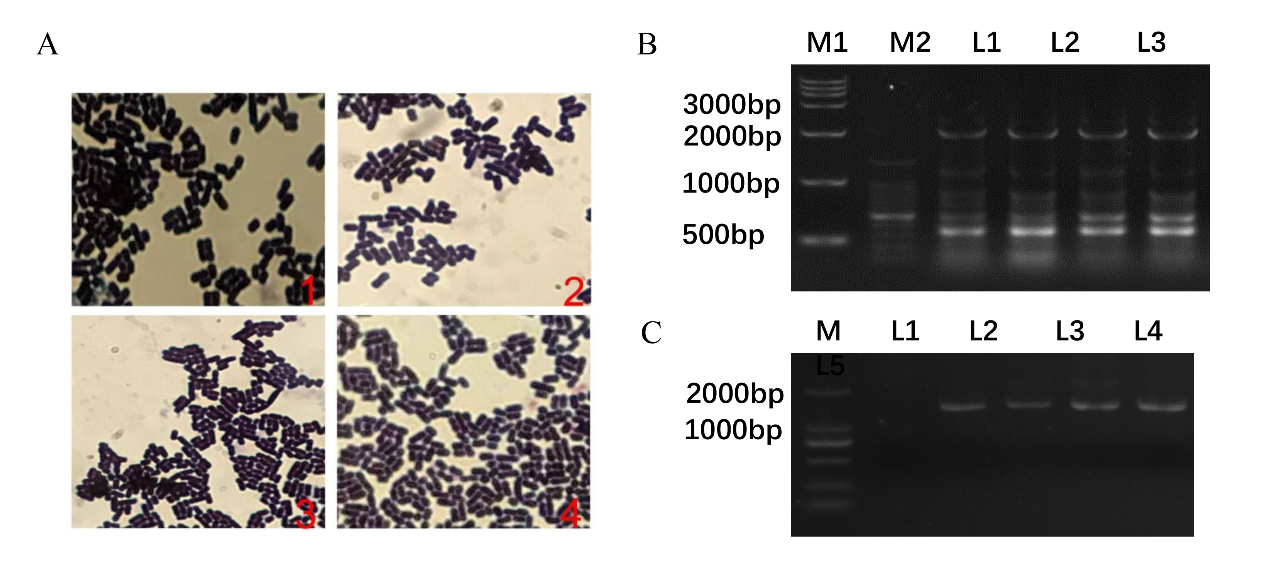


Figure S2. LR-LFCA localization in healthy piglets

The 4-day-old piglets were given oral administration of LR-LFCA for three consecutive days, and the intestinal mucus was taken on the 7th day, 14th day and 21th day after oral administration, the intestinal mucous was homogenized plated on MRS agar plates containing 10 μg/mL chloramphenicol. The plates were incubated overnight at 37°C for 48h and colonies were counted to analyze the colonization ability of LR-LFCA in piglets. Data are presented as the means ± SD. ^*^*p* < 0.05 and ^**^*p* < 0.01 vs. the control group.


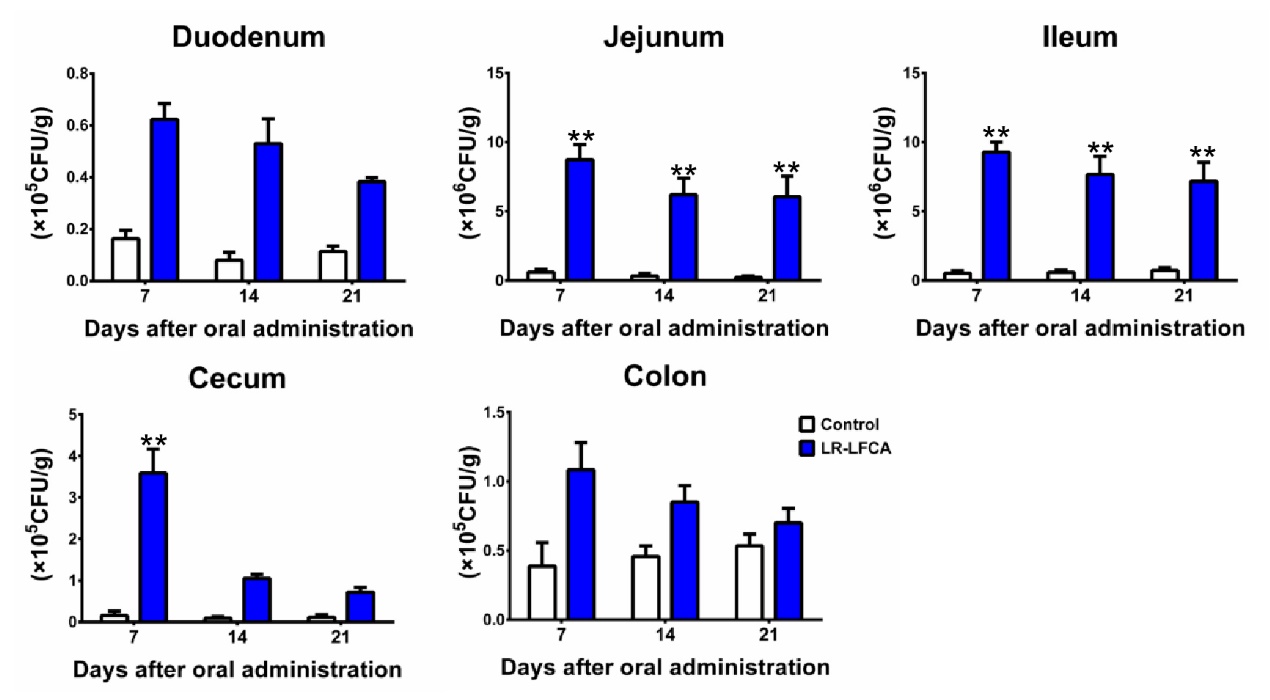


Figure S3. The map of recombinant plasmid pPG612-T7g10-sp-LFCA

Construction diagram of recombinant plasmid pPG612-T7g10-sp-LFCA. HCE strong constitutive promoter; T7g10 transcriptional enhancer; sp signal peptide of peptidoglycan hydrolase; LFCA coding sequence; rrnBT1T2 terminator; replicon C (rep C); replicon A (rep A); chloramphenicol coding sequence (Cm+)


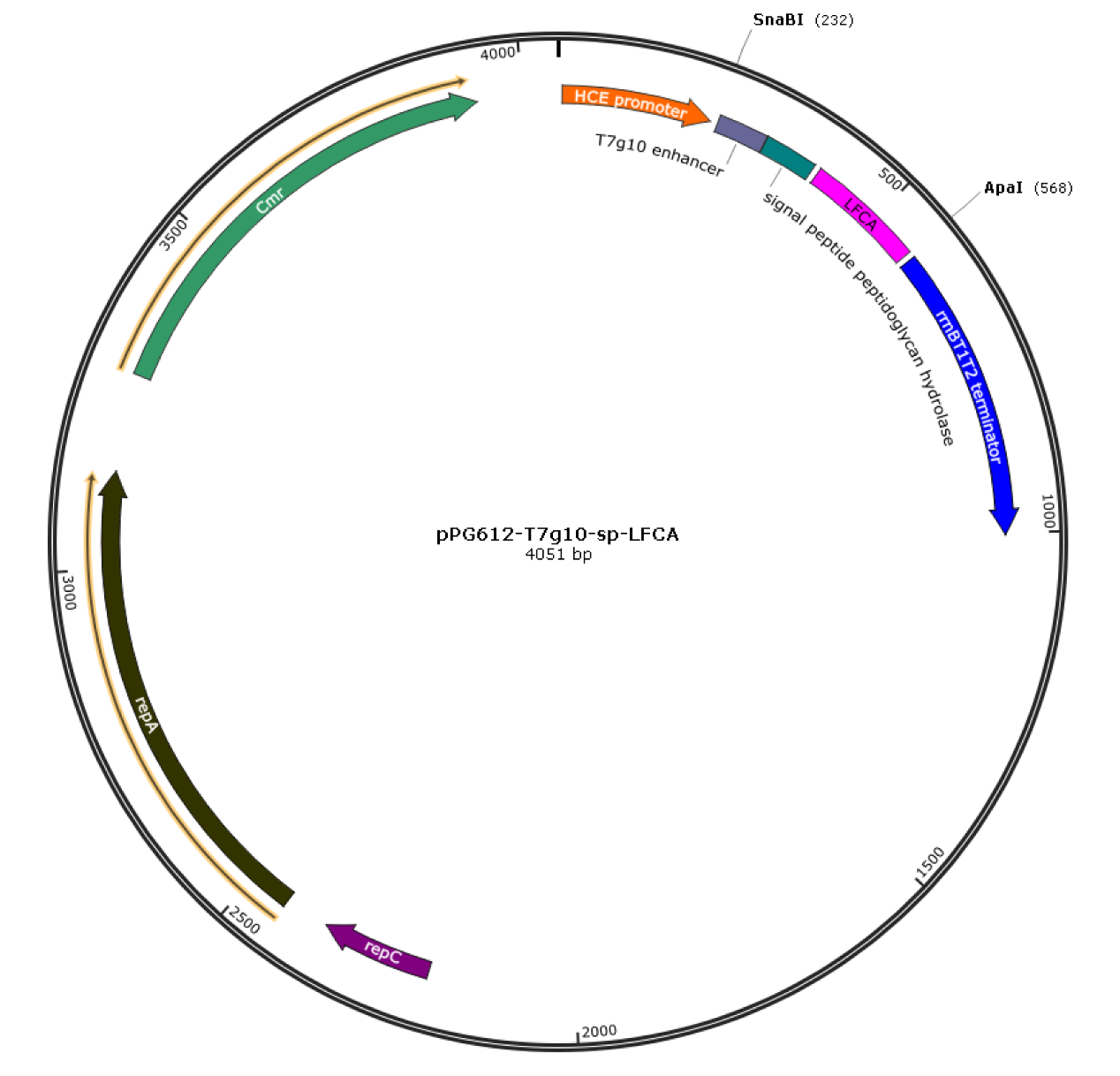

Supplement: Supplemental Material [file KGMI_A_1956281_SM8473.docx]
